# Multimodal predictive factors of metastasis in lymph nodes posterior to the right recurrent laryngeal nerve in papillary thyroid carcinoma

**DOI:** 10.3389/fendo.2023.1187825

**Published:** 2023-07-12

**Authors:** Yi Gong, Zhongkun Zuo, Kui Tang, Yan Xu, Rongsen Zhang, Qiang Peng, Chengcheng Niu

**Affiliations:** ^1^ Department of General Surgery, The Second Xiangya Hospital, Central South University, Changsha, Hunan, China; ^2^ Department of Ultrasound Diagnosis, The Second Xiangya Hospital, Central South University, Changsha, Hunan, China

**Keywords:** contrast-enhanced ultrasound (CEUS), conventional Ultrasound, central cervical lymph node metastasis, papillary thyroid carcinomas (PTCs), lymph nodes posterior to the right recurrent laryngeal nerve metastasis

## Abstract

**Objective:**

The lymph node posterior to the right recurrent laryngeal nerve (LN-prRLN) is a crucial component of the central lymph nodes (LNs). We aimed to evaluate multimodal predictive factors of LN-prRLN metastasis in patients with papillary thyroid carcinomas (PTCs), including the clinical data, pathologic data, and preoperative sonographic characteristics of PTCs.

**Methods:**

A total of 403 diagnosed PTC patients who underwent unilateral, sub-total, or total thyroidectomy with central neck dissection were enrolled in this retrospective study. The clinical data, pathologic data, conventional ultrasound (US) and contrast-enhanced ultrasound (CEUS) characteristics of PTCs were collected and evaluated for predicting LN-prRLN metastasis.

**Results:**

In this study, 96 PTC patients with LN-prRLN metastasis and 307 PTC patients without LN-prRLN metastasis were included. Univariate analysis demonstrated that PTC patients with LN-prRLN metastasis more often had younger age, larger size, multifocal cancers, A/T < 1, well-margins, microcalcification, petal-like calcification, internal vascularity, centripetal perfusion pattern and surrounding ring enhancement. Multivariate logistic regression analysis revealed that the CEUS centripetal perfusion pattern, central LN detected by ultrasound and LN-arRLN metastasis were independent characteristics for predicting LN-prRLN metastasis in PTC patients.

**Conclusion:**

According to our research, it is essential for clinicians to thoroughly dissect central LNs, particularly LN-prRLNs.

## Introduction

PTCs are one of the most prevalent thyroid cancer histological subtypes, with rising incidence and substantial lymph node metastatic rates (1, 2). According to studies, PTC lymph node metastasis cases range from 20% to 90% of total PTC cases, with central lymph node (level VI) metastasis constituting the majority of cases (3). Thyroidectomy and lymph node dissection are the two basic treatments for PTCs (4). Prelaryngeal LNs, pretracheal LNs, left paratracheal LNs, and right paratracheal LNs are the four groups that typically make up cervical central LNs. Due to the anatomical variations of the recurrent laryngeal nerves on both sides, the right paratracheal LNs are divided into those that are anterior to the right recurrent laryngeal nerve (LN-arRLN) and those that are posterior to the right recurrent laryngeal nerve (LN-prRLN) (5, 6).

Due to the difficult surgical technique and the high rate of complications as a result of the deep position with confined exposure, some researchers contend that routine LN-prRLN removal is not necessary. Furthermore, it can be difficult to assess metastases before surgery, and even experienced surgeons are likely to ignore this location. However, the LN-prRLN metastasis rate in PTC patients has been reported to be as high as 2.7–30.4% (3, 5, 7). The first surgical treatment strategy for LN-prRLN is crucial for the patient’s prognosis. Inadequate cervical LN dissection may increase the risk of recurrence and complications due to repeated operations, so some researchers have suggested routine removal of LN-prRLNs to maximize treatment success (8). These disagreements demonstrate that LN-prRLN merits consideration and additional study. Hence, it is critical to appropriately estimate the probability of LN-prRLN metastasis, which could aid in determining the scope of surgery and lowering the recurrence rates.

Some studies have reported the clinicopathologic risk factors for LN-prRLN metastasis for PTCs. Zhang et al. reported that tumor size larger than 1 cm, multifocality, and extrathyroidal extension were independent predictors of LN-prRLN metastasis in right-sided PTC (3). Zhou et al. proved that tumor size larger than 1. 5cm, extrathyroidal extension, and LN-arRLN metastasis were independent predictors of LN-prRLN metastasis in cN0 PTC (5). However, the research solely included clinicopathologic features, limiting their clinical application; thus, the preoperative sonographic features of primary tumors are essential for evaluating the risk factors for LN-prRLN metastasis in PTCs. US is an incredibly sensitive tool for evaluating initial PTC lesions, providing ultrasonographic signs of PTCs that can be utilized to predict cervical LN metastases in PTC patients and offer a basis for the selection of surgical techniques (9, 10). Unfortunately, few similar studies have been conducted in LN-prRLN metastasis (11).

CEUS, which provides more information about vascularity than conventional US, could reveal the thyroid nodule’s microvasculature and increase the diagnostic accuracy of thyroid nodules (12, 13). Our previous studies found that preoperative CEUS characteristics could offer help in predicting central lymph node metastasis in PTCs with coexistent Hashimoto’s thyroiditis and identifying malignant cervical LNs from benign cervical LNs (14–16).

By analyzing the preoperative US characteristics of PTCs and the postoperative pathological data in 403 PTC patients, this study seeks to identify potential predictors for the preoperative clinical evaluation of LN-prRLN metastases.

## Methods and materials

### Patients

The study was approved by the Ethical Committee of the Second Xiangya Hospital of Central South University in China and performed in accordance with the Declaration of Helsinki for human study. The requirement of informed consent from human subjects was waived by IRBs due to the retrospective nature of the review of images acquired for clinical diagnostic purposes. From May 2021 to August 2022, 1253 consecutive patients with thyroid nodules who received conventional US and CEUS examinations were prospectively enrolled in this single-center study. The inclusion criteria were as follows: (i) patients with thyroid nodules who underwent conventional US and CEUS examinations and (ii) pathological examination confirming PTCs after surgery. Patients with the following criteria were excluded: (i) a history of neck surgery or neck radiotherapy; (ii) central cervical lymph node dissection not performed; and (iii) LN-prRLN without clear pathological diagnosis. The final diagnosis of malignant thyroid nodules and metastatic lymph nodes was confirmed by histopathology after surgery. For patients with multifocal PTCs, the largest one was selected for CEUS examination. Finally, 403 PTC patients (96 patients with LN-prRLN metastasis and 307 patients without LN-prRLN metastasis proven by pathological examination) were included in this study. In addition, TSH, thyroid peroxidase antibody (A-TPO), and thyroglobulin antibody (A-TG) levels were evaluated in all patients within one week before surgery.

### Conventional US and CEUS examination

A GE E11 US scanner with a 3-12L (3-12 MHz) linear array transducer or Siemens Acuson S3000 US scanner with a 9L4 (4–9 MHz) linear array transducer was used for conventional US and CEUS examination. All examinations were performed by operators with more than ten years of experience in thyroid ultrasound diagnosis and five years of experience in performing CEUS examinations. Two other investigators with 5 years of experience in thyroid ultrasound diagnosis analyzed the US images independently. Disagreements were solved by consensus. Two-milliliters of SonoVue (Bracco, Italy) or 0.4 mL of Sonozoid (GE, USA) was injected intravenously, followed by a saline flush of 5 mL. Thyroid nodule imaging lasted at least 30 seconds.

Conventional US features of the thyroid nodules were classified as follows (13): shape (taller than wide, A/T >1; or wider than tall, A/T< 1); margins (well-defined or ill-defined); echogenicity (marked hypo-, hypo-, iso- or hyper-echoic); calcification (no calcification, microcalcification<1 mm in diameter, macrocalcification >1 mm in diameter); petal-like calcification (defined as microcalcification displayed around the thyroid nodule like a petal) (17) and internal vascularity (present or absent).

The following categories describe the thyroid nodules’ CEUS characteristics (14): enhancement type (hyper-, iso- or hypo-enhancement); enhancement uniformity (heterogeneous or homogeneous enhancement); perfusion pattern (centripetal perfusion, the perfusion of microbubbles from the periphery to the center; centrifugal perfusion, the perfusion of microbubbles from the center to the periphery); and surrounding ring enhancement (presence or absence).

### Surgical treatment proposal

Three surgical options (unilateral, sub-total, or total thyroidectomy with central neck dissection) are available, according to the Chinese Thyroid Association’s guidelines. The procedure was chosen based on the surgeon’s desire and a comprehensive analysis of the patient’s condition. Prelaryngeal LN, pretracheal LN, left paratracheal LN, LN-arRLN, and LN-prRLN were the five subgroups of the central LNs and sent for individual pathological evaluation. If the lateral cervical LNs were diagnosed as metastatic LNs from PTCs, lateral cervical lymph node dissection was performed.

### Reference standard

The histopathological results after surgery were used as the only reference standard for the final diagnosis of malignant thyroid nodules and metastatic lymph nodes.

### Statistical analysis

The statistical analysis was carried out using SPSS version 21.0. (SPSS, Chicago, IL, USA). The mean and standard deviation (SD) of continuous data were displayed, and they were compared using an independent t test. Categorical data were evaluated using the chi-square test and are shown as percentages. To evaluate multimodal factors and their independent associations with LN-prRLN metastasis, binary logistic regression was performed. P < 0.05 was used to evaluate whether differences were statistically significant.

## Results

This study comprised a total of 403 PTC patients, including 307 individuals without LN-prRLN metastasis and 96 patients with LN-prRLN metastasis. **Table 1** displays the clinical and pathologic characteristics of the patients. Among the 403 patients with PTCs, the mean age was 41.69 ± 12.01 years (range 18-73 years), 57.8% (233/403) were younger than 45 years, and 75.4% (304/403) were female patients. The mean tumor size was 12.56 ± 8.65 mm (range 2–51 mm). In total, 32.3% (130/403) of patients had Hashimoto’s thyroiditis, 46.2% (186/403) had multifocal PTCs, 51.9% (209/403) had lesions in the right lobe, 9.9% (40/403) had lesions in the left lobe, 2.5% (10/403) had lesions in the isthmus, and 35.7% (144/403) had lesions in the bilateral lobe. Level VI lymph nodes were detected in 176 (43.7%) patients on preoperative conventional US examination. Sub-total and total thyroidectomy with central neck dissection were performed in 367 (91.1%) patients, and unilateral thyroidectomy with central neck dissection was performed in 36 (8.9%). Histopathological examination showed that the rates of left paratracheal LN, prelaryngeal LN, pretracheal LN, LN-arRLN, LN-prRLN and lateral LN metastasis were 28.5% (115/403), 13.2% (53/403), 30.0% (121/403), 36.0% (145/403), 23.8% (96/403) and 24.6% (99/403), respectively. In addition, among patients without any other cervical lymph node metastasis, the rate of LN-prRLN metastasis was 8.2% (8/96). Transient and permanent hypoparathyroidism were noted in 55 (13.6%) and 2 (0.5%) patients, respectively. Voice change developed in 10 (2.5%) patients, but all patients recovered during follow-up, and no patient had consistent dysfunction with vocal cord paralysis.

**Table 1:** Clinical and pathologic data of the enrolled 403 patients.

Variables	Numbers (%)
Age (year)	41.69 ± 12.01
< 45	233 (57.8%)
≥ 45	170 (42.2%)
Gender	
Male	99 (24.6%)
Female	304 (75.4%)
Tumor size (mm)	12.56 ± 8.65
≤ 5 mm	62 (15.4%)
>5 and ≤10 mm	163 (40.4%)
>10 and ≤20 mm	114 (28.3%)
>20 and ≤40 mm	56 (13.9%)
> 40 mm	8 (2.0%)
Coexistent Hashimoto’s thyroiditis	
Yes	130 (32.3%)
No	273 (67.7%)
Multifocality	
Yes	186 (46.2%)
No	217 (53.8%)
Tumor location	
Right lobe	209 (51.9%)
Left lobe	40 (9.9%)
Isthmus	10 (2.5%)
Bilateral lobe	144 (35.7%)
Central LN detected by Ultrasound**	176 (43.7%)
Operation type##	
TT or ST + central neck dissection	367 (91.1%)
UL + central neck dissection	36 (8.9%)
LN metastatic sites	
Left paratracheal LN	115 (28.5%)
Prelaryngeal LN	53 (13.2%)
Pretracheal LN	121 (30.0%)
LN-arRLN&	145 (36.0%)
LN-prRLN#	96 (23.8%)
Lateral LN	99 (24.6%)
Hypoparathyroidism	
Transient	55 (13.6%)
Permanent	2 (0.5%)
Vocal cord paralysis	
Transient	10 (2.5%)
Permanent	0 (0.0%)

**LN, lymph node.

##TT, total thyroidectomy; ST, sub-total thyroidectomy; UL, unilateral lobectomy.

&LN-arRLN, lymph node anterior to the right recurrent laryngeal nerve.

#LN-prRLN, lymph node posterior to the right recurrent laryngeal nerve.

The univariate analysis of predictors for LN-prRLN is reported in **Table 2**. The average age of PTC patients with or without LN-prRLN metastasis was 36.65 ± 12.19 years (range: 18-71 years) or 43.27 ± 11.52 years (range: 18-73 years), respectively. Seventy-two (75.0%) patients with LN-prRLN metastasis were younger than 45 years, and 161 (52.4%) patients without LN-prRLN metastasis were younger than 45 years (p = 0.000), showing that the patients with LN-prRLN metastasis were more inclined to be younger. The mean diameters of PTCs with or without LN-prRLN metastasis were 18.23 ± 10.94 mm (range: 3-51 mm) and 10.79 ± 6.93 mm (range: 2-42 mm), respectively, showing that the former had considerably higher mean diameters than the latter (p = 0.000). A size > 10 mm was present in 68 (70.8%) of the patients with LN-prRLN metastasis and 110 (35.8%) of the patients without LN-prRLN metastasis (p = 0.000). Multifocal malignancies were present in 53 (55.2%) individuals with LN-prRLN metastases and in 133 (43.3%) patients without this occurrence (p = 0.041). Only the largest PTC was selected in patients with multifocal PTCs. LN-prRLN metastasis did not appear to be correlated with any other variables (all p>0.05).

**Table 2:** Univariate analysis of predictor for lymph node posterior to the right recurrent nerve metastasis (LN-prRLN).

Variables	LN-prRLN status		P valve
	Positive (n=96)	Negative (n=307)	
Age (year)	36.65 ± 12.19	43.27 ± 11.52	0.000*
< 45	72 (75.0%)	161 (52.4%)	0.000*
≥ 45	24 (25.0%)	146 (47.6%)	
Gender			0.141
Male	29 (30.2%)	70 (22.8%)	
Female	67 (69.8%)	237 (77.2%)	
Tumor size (mm)	18.23 ± 10.94	10.79 ± 6.93	0.000*
≤ 10 mm	28 (29.2%)	197 (64.2%)	0.000*
> 10 mm	68 (70.8%)	110 (35.8%)	
Coexistent Hashimoto’s thyroiditis			0.135
Yes	25 (26.0%)	105 (34.2%)	
No	71 (74.0%)	202 (65.8%)	
Multifocality			0.041*
Yes	53 (55.2%)	133 (43.3%)	
No	43 (44.8%)	174 (56.7%)	
Tumor location			0.081
Right lobe	52 (54.2%)	157 (51.1%)	
Left lobe	3 (3.1%)	37 (12.1%)	
Isthmus	2 (2.1%)	8 (2.6%)	
Bilateral lobe	39 (40.6%)	105 (34.2%)	
A/T			0.000*
> 1	45 (46.9%)	207 (67.4%)	
< 1	51 (53.1%)	100 (32.6%)	
Margin			0.009*
Well-defined	13 (13.5%)	17 (5.5%)	
Ill-defined	83 (86.5%)	290 (94.5%)	
Echogenicity			0.359
Marked hypoechoic or hypoechoic	93 (96.9%)	302 (98.4%)	
Iso- or hyperechoic	3 (3.1%)	5 (1.6%)	
Calcification			0.001*
Absent or macrocalcification	6 (6.2%)	65 (21.2%)	
Microcalcification	90 (93.8%)	242 (78.8%)	
Petal-like calcification			0.000*
Present	19 (19.8%)	22 (7.2%)	
Absent	77 (80.2%)	285 (92.8%)	
Internal vascularity			0.000*
Yes	26 (27.1%)	29 (9.4%)	
No	70 (72.9%)	278 (90.6%)	
Enhancement type			0.060
Hyper- or iso-enhancement	26 (27.1%)	56 (18.2%)	
Hypo-enhancement	70 (72.9%)	251 (81.8%)	
Perfusion pattern			0.000*
Centrifugal	34 (35.4%)	32 (10.4%)	
Centripetal	62 (64.6%)	275 (89.6%)	
Enhancement uniformity			0.703
Heterogeneous enhancement	85 (88.5%)	276 (89.9%)	
Homogeneous enhancement	11 (11.5%)	31 (10.1%)	
Surrounding ring enhancement			0.014*
Present	9 (9.4%)	10 (3.3%)	
Absent	87 (90.6%)	297 (96.7%)	
Central LN detected by Ultrasound**			0.000*
Yes	74 (77.1%)	102 (33.2%)	
No	22 (22.9%)	205 (66.8%)	
LN metastatic sites			
Left paratracheal LN	51 (53.1%)	64 (20.8%)	0.000*
Prelaryngeal LN	28 (29.2%)	25 (8.1%)	0.000*
Pretracheal LN	53 (55.2%)	68 (22.1%)	0.000*
LN-arRLN&	80 (83.3%)	65 (21.2%)	0.000*
Lateral LN	57 (59.4%)	42 (13.7%)	0.000*

**LN, lymph node.

&LN-arRLN, lymph node anterior to the right recurrent laryngeal nerve.

*p < 0.05 was considered a significant difference.

**Figure 1:**
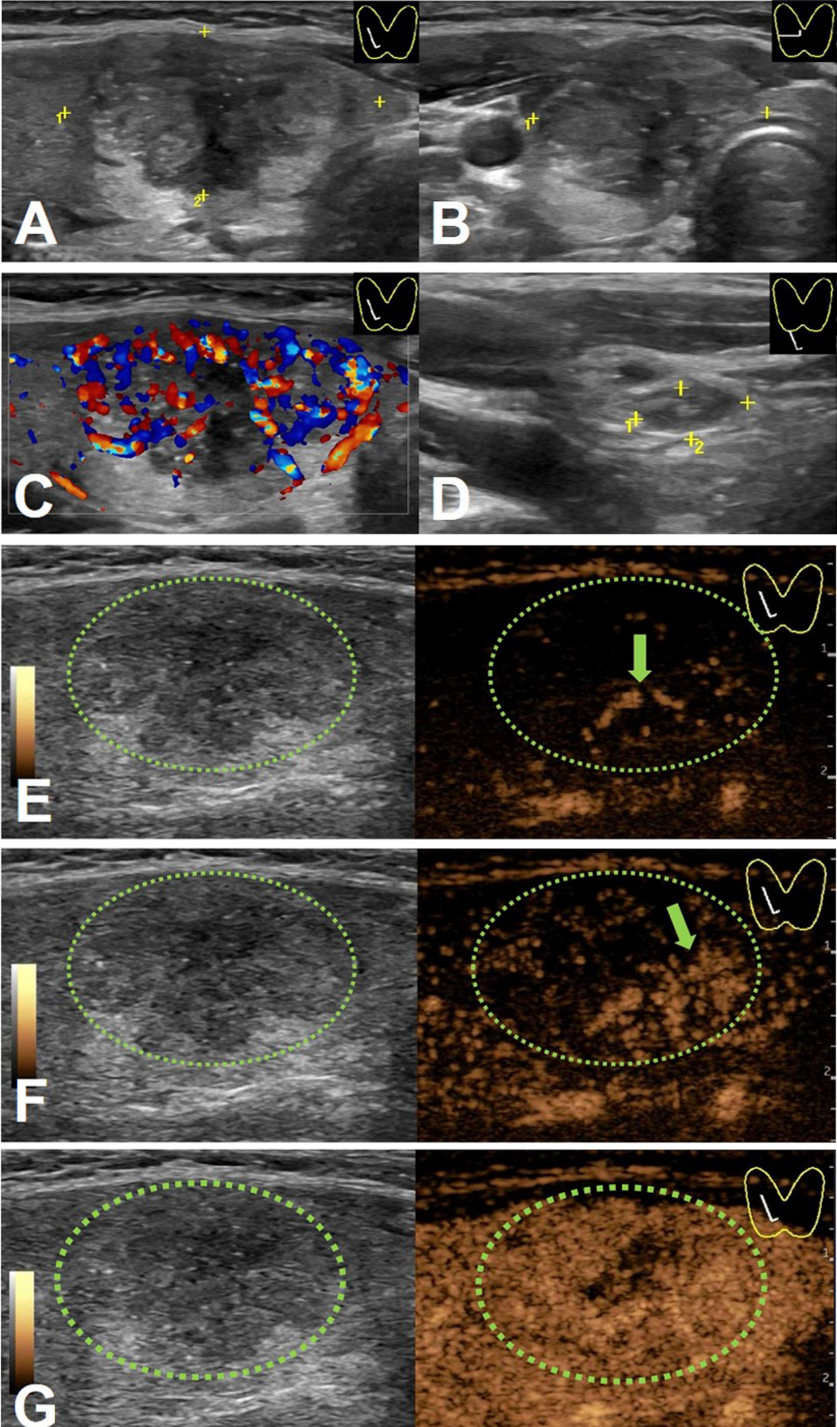
An 18-y-old female PTC patient with LN-prRLN metastasis. Initial longitudinal scan **(A)** and transverse scan **(B)** showing a 30-mm thyroid nodule with hypo-echogenicity, ill-defined margins, and petal calcification. **(C)** Doppler image revealing abundant signals in the nodule. **(D)** A level VI lymph node (yellow cross) identified on conventional US. **(E)** CEUS image 6 s after injection of contrast-enhanced agent, the thyroid nodule enhanced from the center to the periphery (green arrow). **(F)** CEUS image 7 s after injection of contrast-enhanced agent, the thyroid nodule showed heterogeneous enhancement (green arrow). **(G)** CEUS image 8 s after injection of contrast-enhanced agent, the thyroid nodule showed heterogeneous iso-enhancement.

**Figure 2:**
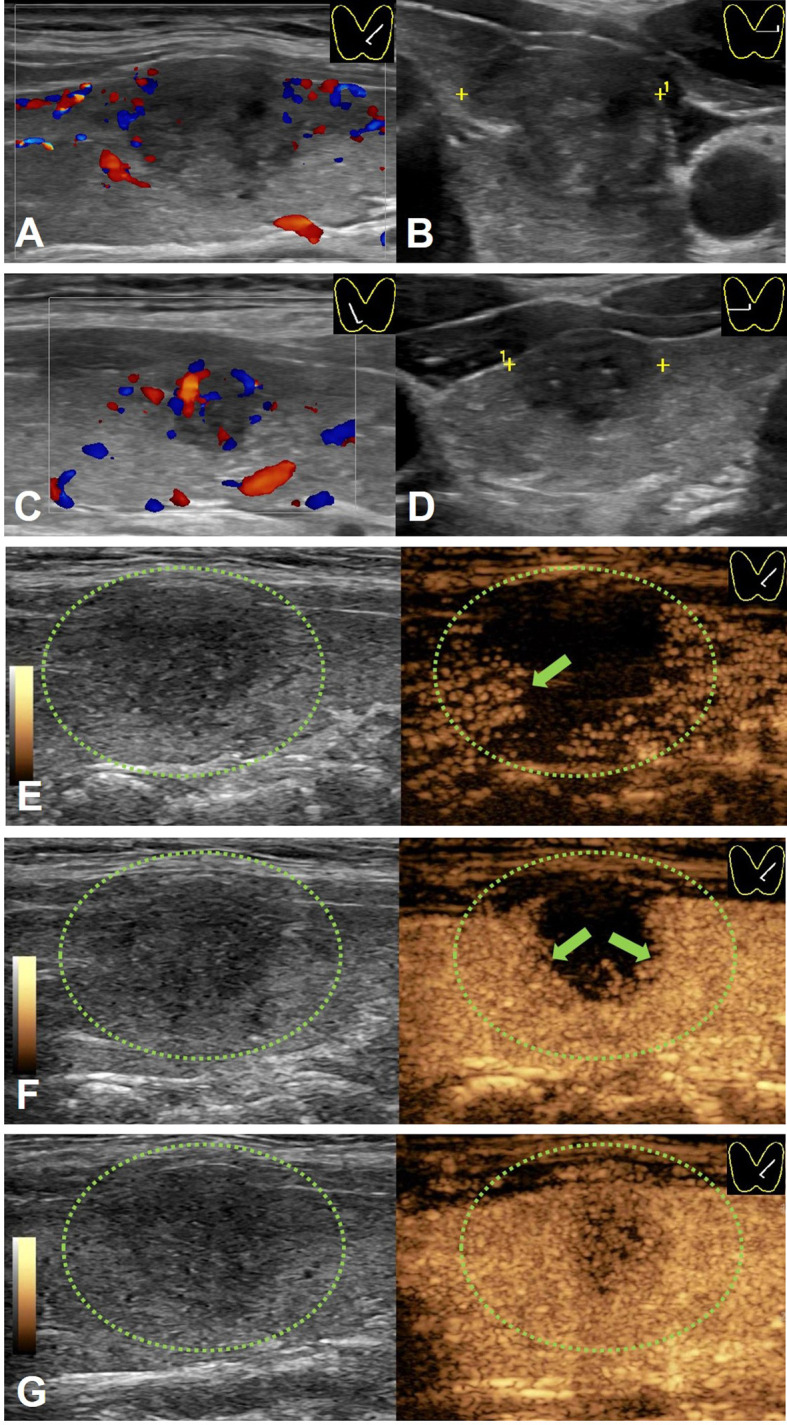
A 41-y-old male PTC patient without LN-prRLN metastasis. **(A)** Longitudinal Doppler image and **(B)** transverse scan image showing a 17-mm thyroid nodule with hypo-echogenicity, ill-defined margins, microcalcification and poor signals in the nodule in the left lobe. **(C)** Longitudinal Doppler image and **(D)** transverse scan image showing a 11-mm thyroid nodule with hypo-echogenicity, ill-defined margins, microcalcification and few signals in the nodule in the right lobe. **(E)** CEUS image 10 s after injection of contrast-enhanced agent, the thyroid nodule enhanced from the periphery to the center (green arrow). **(F)** CEUS image 12 s after injection of contrast-enhanced agent, the thyroid nodule showed heterogeneous enhancement (green arrow). **(G)** CEUS image 14 s after injection of contrast-enhanced agent, the thyroid nodule showed heterogeneous hypo-enhancement.

Compared with PTCs without LN-prRLN metastasis, PTCs with LN-prRLN metastasis had more A/T < 1 and well-defined margins, microcalcification, petal-like calcification and internal vascularity on conventional US images. For CEUS images, the PTCs with LN-prRLN metastasis showed a more centrifugal perfusion pattern and the presence of surrounding ring enhancement than the PTCs without LN-prRLN metastasis (**Figures 1**, **2**).

Level VI lymph nodes were detected in 74 (77.1%) patients in the PTC with LN-prRLN metastasis group compared with 102 (33.2%) patients in the PTC without LN-prRLN metastasis group on preoperative conventional US examination. In addition, all metastatic rates of left paratracheal LNs, prelaryngeal LNs, pretracheal LNs, LN-arRLNs, LN-prRLNs and lateral LNs were higher than those of the PTCs without LN-prRLN metastasis group on histopathological examination.

All of the statistically significant factors (p < 0.05) underwent multivariate logistic regression analysis. The outcomes showed that the CEUS centrifugal perfusion pattern (B= 1.314, OR = 3.720, 95% CI = 1.808-7.654, p=0.000), central LN detected by ultrasound (B= 1.027, OR = 2.792, 95% CI = 1.440-5.415, p=0.002), and LN-arRLN metastasis (B =0.753, OR =2.123, 95% CI = 1.083-4.163, p=0.028) were independent characteristics for predicting LN-prRLN metastasis (**Table 3**).

**Table 3:** Multivariate analysis of the predictors of lymph node posterior to the right recurrent laryngeal nerve metastasis (LN-prRLN).

Variables	B	SE	Odd ratio	P valve	95% CI
Centrifugal perfusion	1.314	0.368	3.720	0.000*	1.808-7.654
Central LN detected by Ultrasound**	1.027	0.338	2.792	0.002*	1.440-5.415
LN-arRLN metastasis&	0.753	0.344	2.123	0.028*	1.083-4.163
Constant	-3.452	0.330	0.032	0.000*	

**LN, lymph node.

&LN-arRLN, lymph node anterior to the right recurrent laryngeal nerve.

*p < 0.05 was considered a significant difference.

## Discussion

Currently, there is still controversy about whether to dissect the LN-prRLN in the absence of certain LNs that are positive. Dissection of the LN-prRLN requires patience and experience and could lead to nerve damage and parathyroid injury, affecting the patient’s quality of life (18). According to some researchers, PTCs are a kind of tumor; hence, LN-prRLN dissection is not needed, thus reducing surgical risks to patients after surgery. Hypoparathyroidism and vocal cord paralysis were the two serious complications after thyroid surgery. Transient and permanent hypoparathyroidism occurred in 4.1-51.9% and 0-1.1% of patients, respectively (6, 18–20). Postoperative voice change was found in 4.9-7.4% of patients, while consistent voice change with vocal cord immobility was found in 0-4.0% of patients (6, 18–20).

Although PTCs have a favorable prognosis, other scholars contend that the central LNs are the most frequent sites for lymph node metastasis with a high incidence of 13.7%-72.2% (14, 16, 21). Thorough initial dissection with simultaneous dissection of the LN-prRLN is essential to reduce local recurrence and metastasis and avoid significant complication rates by secondary surgery. Secondary thyroid surgery is difficult for young and inexperienced surgeons, but even skilled and experienced surgeons can have difficulty in mapping the recurrent laryngeal nerve and preventing inadvertent recurrent laryngeal nerve injury, particularly in nonanatomical fields with many scars from the first operations (22, 23). In accordance with the recommendations of the American Thyroid Association (ATA), cervical LNs should be evaluated using preoperative neck US to determine the surgical approach, particularly for lymph node dissection (4). However, conventional US only has a sensitivity of less than 30% for central LNs (24, 25). The risk factors for LN-prRLN metastases in PTCs must be identified for surgeons to choose the best surgical procedures. The variables that predict LN-prRLN metastases in PTC patients, however, are not well known.

Univariate analysis in this study demonstrated that age < 45 years was a clinical risk factor related to LN-prRLN metastasis in PTC patients, demonstrating that patients with LN-prRLN metastasis were more likely to be young people, which was consistent with other previous studies (11, 26). In addition, tumor size > 10 mm was also a clinical risk factor associated with LN-prRLN metastasis, indicating that tumor size was closely linked to an elevated risk of LN-prRLN metastasis, which was also consistent with other previous reports (11, 19).

Recent studies have shown that US characteristics could help predict cervical central LN metastasis in PTC patients (9, 10, 27). Xu et al. reported that a virtual touch tissue imaging area ratio >1, abnormal cervical LNs, microcalcification and multiple nodules were risk factors for the prediction of cervical central LN metastasis in PTC patients (10). Liu et al. found that microcalcification, resistance index >0.7 and multiple nodules have predictive value in central LN metastasis of PTCs (27). However, few studies have been reported for LN-prRLN metastasis prediction by ultrasonographic features. In this study, we found that multiple nodules, calcification, petal-like calcification and internal vascularity were significantly associated with LN-prRLN metastasis in PTC patients. However, the suspicious ultrasonographic features for malignant thyroid nodules, including a taller-than-wider shape (A/T <1) and an ill-defined margin were protective factors for LN-prRLN metastasis in PTC patients, which may contribute to the tumor size of thyroid nodules. The smaller thyroid nodules in PTC patients without LN-prRLN metastasis were more likely to have an ill-defined margin and a taller-than-wider shape of PTCs, which was also reported in our previous study on PTCs with HT patients with coexisting central LN metastasis (14).

Compared to color Doppler technology, the accuracy of thyroid nodule diagnosis may improve with the use of CEUS, which can detect more tumor microvessels. However, to the best of our knowledge, these CEUS parameters for predicting LN-prRLN metastasis in PTC patients have not been explored to date. In our study, we found that the centrifugal perfusion pattern and presence of surrounding ring enhancement were significantly associated with LN-prRLN metastasis in PTC patients. Multivariate analysis demonstrated that the centrifugal perfusion pattern was one of the independent characteristics for the presence of LN-prRLN metastasis.

Although central LNs have a low display rate on preoperative conventional US examination, the present study found central LNs in 176 (43.7%) patients. Moreover, the display rate of central LNs in the PTC with LN-prRLN metastasis group was as high as 77.1%, which was significantly higher than that of 33.2% in PTC without LN-prRLN metastasis group. Multivariate analysis demonstrated that central LN detected by US was another independent characteristic for the prediction of LN-prRLN metastasis, indicating that the centrifugal perfusion pattern on CEUS and central LN detected by US could serve as preoperative factors for predicting LN-prRLN metastasis. In addition, all histopathological metastatic rates of left paratracheal LNs, prelaryngeal LNs, pretracheal LNs, LN-arRLNs, LN-prRLNs and lateral LNs were significantly higher than those of PTCs without LN-prRLN metastasis. In this study, individuals who had LN-arRLN metastases had a nearly fourfold increased probability of LN-prRLN metastasis than patients who did not. According to the multivariate analysis, LN-arRLN metastasis in PTC patients was a significant risk factor for LN-prRLN metastasis, suggesting that the LN-arRLN compartment may be used intraoperatively to predict LN-prRLN metastasis.

According to the findings of univariate and multivariate analyses, the centrifugal perfusion pattern on CEUS, central LN detected by US, and LN-arRLN metastasis were independent predictors of LN-prRLN metastasis in these patients. In that case, suggesting that the LN-prRLN should be removed with thorough neck dissection to prevent metastasis.

There are several limitations to our study. First, it was a single institutional retrospective analysis, hence the findings may not have been applicable to other PTC patient populations. Second, selection bias was also present. To further explain these findings, a large-scale prospective multicenter investigation is needed.

## Conclusions

In summary, complete central LN dissection should be taken into consideration given the rate of LN-prRLN metastasis and the challenges and complications of reoperation. Centrifugal perfusion patterns on CEUS, central LN detected by US and LN-arRLN metastasis are helpful in predicting metastasis to LN-prRLNs. Thus, preoperative conventional US and CEUS may serve as useful tools in predicting LN-prRLN metastasis in PTC patients.

## Data availability statement

The original contributions presented in the study are included in the article/supplementary material. Further inquiries can be directed to the corresponding author.

## Ethics statement

The studies involving human participants were reviewed and approved by the Ethics Committee of Second Xiangya Hospital, Central South University, China. The patients/participants provided their written informed consent to participate in this study. Written informed consent was obtained from the individual(s) for the publication of any potentially identifiable images or data included in this article.

## Author contributions

CN contributed to the conception and design of the work. YG participated to data analysis and manuscript writing. ZZ, KT, YX, RZ and QP participated to data collection and patients’ follow-up. All authors contributed to the article and approved the submitted version.
